# Self-Esteem as a Predictor of Dental Anxiety in Turkish Children: A Cross-Sectional Study

**DOI:** 10.3390/children13081102

**Published:** 2026-08-18

**Authors:** Aybike Memiş, Yasemin Akın, Merve Demirkoparan, Didem Atabek

**Affiliations:** 1 Department of Pediatric Dentistry, Faculty of Dentistry, Aksaray University, 68000 Aksaray, Turkey; 2 Department of Pediatric Dentistry, Faculty of Dentistry, Gazi University, 06490 Ankara, Turkey

**Keywords:** dental anxiety, self-esteem, behavioral management

## Abstract

**Highlights:**

**What are the main findings?**

- Self-esteem was the only significant independent predictor of dental anxiety (r = −0.301, *p* < 0.001), while age, gender, and socioeconomic status showed no association.
- Dental anxiety affected 24.5% of the 204 children studied, and lower self-esteem was associated with higher anxiety, though the variance explained remained modest (Adjusted R^2^ = 0.080).

**What are the implications of the main findings?**

- Self-esteem emerges as a relevant factor in pediatric dental anxiety assessment, beyond standard demographic factors.
- Considering children’s psychological profile in behavioral management could support existing approaches, though this needs further study.

**Abstract:**

**Background**: Dental anxiety is a prevalent condition in pediatric oral healthcare and linked with multiple etiologic factors. Although sociodemographic factors are frequently examined, the influence of internal psychological constructs such as self-esteem remains underexplored in children. This cross-sectional study aims to evaluate the association between dental anxiety and self-esteem among school-age Turkish children. **Methods**: The study was conducted with 204 participants aged 7–12 years. Dental anxiety and self-esteem were assessed using Corah’s Dental Anxiety Scale and the Rosenberg Self-Esteem Scale, respectively. Group comparisons were performed with the Mann–Whitney U and Kruskal–Wallis tests, and their association was examined with Spearman’s rank correlation. Independent predictors of dental anxiety were further identified through a stepwise multiple linear regression. **Results**: Dental anxiety was prevalent in 24.5% of participants. No significant differences in anxiety or self-esteem were found by gender, age, or socioeconomic status (*p* > 0.05). A moderate negative correlation (r = −0.301, *p* < 0.001) was observed between self-esteem and dental anxiety. Regression analysis confirmed self-esteem as the only significant independent predictor of dental anxiety (Adjusted R^2^ = 0.080, *p* < 0.001) in this model. **Conclusions**: Findings indicate that self-esteem is independently associated with pediatric dental anxiety, although the explained variance was modest. Whether incorporating children’s psychological profile into behavioral management improves clinical outcomes remains to be investigated.

## 1. Introduction

Dental anxiety refers to feelings of discomfort, physical symptoms, or avoidance behaviors that arise during dental visits or procedures [1]. While it can occur at any age, it is particularly common in children and adolescents. Reported prevalence estimates for dental anxiety in children range from 5.7% to 23.9% [2,3,4]. Dental anxiety has been linked to a range of individual, social, and cultural factors, reflecting its multifactorial etiology [5]. Contributing factors reported in the literature include age, sex, psychological traits, previous traumatic dental or medical experiences, socioeconomic status, and parental dental anxiety [4,6,7,8].

Dental anxiety interferes with children’s access to effective dental care, often leading to postponement or avoidance of treatment and subsequent oral health decline [1,5,9]. This creates a ‘vicious cycle’ in which fear of treatment is continually reinforced [9,10,11,12,13]. Beyond its clinical consequences, oral health also influences children’s psychosocial well-being and quality of life [14]. Those who feel insecure about their dental appearance tend to smile less often, which may adversely affect their self-image, social functioning, and interpersonal relationships [15]. Given its behavioral and clinical implications, understanding the psychological determinants of dental anxiety is crucial for improving pediatric dental care.

Among the psychological determinants of dental anxiety, one factor that has gained increasing attention as a potential mediating influence is self-esteem; it refers to a person’s sense of self-worth or satisfaction with oneself [14] and represents a core component of the developing self-concept during childhood and adolescence [16]. It has been strongly related to psychological, physical, and social well-being [16,17]. Previous studies have reported a positive link between oral health status and self-esteem [14,18]. Oral conditions such as malocclusions, anterior dental trauma, tooth loss, and untreated caries negatively affect self-esteem because of their impact on aesthetic perception and psychosocial well-being [18]. Self-esteem has also been linked to broader psychosocial outcomes, including poorer coping with social stressors such as peer bullying [18] and positive associations with academic achievement [19,20]. It has further been proposed, alongside related constructs such as self-efficacy and self-control, as a determinant of oral health-related behaviors [21].

Although a unified theoretical model linking self-esteem and dental anxiety has yet to be established in the pediatric literature, self-esteem is broadly understood to shape how individuals appraise and cope with stressful events, consistent with its proposed role as a psychological buffer against anxiety [22]. This relationship may be explained by interrelated psychological mechanisms: dental anxiety may lead to poorer oral health, which in turn causes aesthetic and psychosocial dissatisfaction, further undermining self-esteem, while children with lower self-esteem may be less capable of coping with negative emotions, thereby amplifying their anxiety and avoidance responses [14,19]; consistent with this, high dental anxiety has also been associated with reduced self-efficacy [23]. Evidence for a direct association, however, remains limited and inconsistent: a systematic review proposed a conceptual pathway—dental anxiety linked to reduced oral-health-related quality of life, which was in turn associated with lower self-esteem—but could not confirm a direct association between dental anxiety and self-esteem due to insufficient evidence [14], while a cross-cultural comparison of Turkish and Finnish preadolescents found only a weak correlation after adjusting for maternal and psychological covariates [24]. Notably, these studies relied primarily on bivariate correlation and did not control for the simultaneous contribution of sociodemographic variables.

Self-esteem was selected as the primary predictor in this study given its established role in oral health-related behavior [21] and its proposed, though unconfirmed, direct association with dental anxiety [14,19]. This study aims to determine whether self-esteem remains an independent correlate of dental anxiety once sociodemographic factors are accounted for, in a school-aged Turkish population. Self-esteem was hypothesized to be an independent correlate of dental anxiety.

## 2. Materials and Methods

### 2.1. Study Design

This cross-sectional investigation was carried out at the Department of Pediatric Dentistry, Gazi University (Ankara, Turkey) between May and August 2024. The study was approved by the Institutional Ethics Committee (approval number 2024-805) and was conducted in accordance with the Declaration of Helsinki. Participants were 204 children aged 7–12 years. Parents or legal guardians were informed of the study’s objectives and methodology and provided written informed consent; the study procedures were also explained verbally to the child participants in age-appropriate language, and verbal assent was obtained from each child. All participants were informed that they could withdraw from the research at any time without providing a reason. The study followed the STROBE guidelines.

### 2.2. Sample Size Calculation

To determine the sample size, a power analysis was performed using the “G*Power-3.1.9.2” software (Franz Faul, Universität Kiel, Kiel, Germany), yielding a minimum of 138 participants. This was based on a 95% confidence level (α = 0.05) and a theoretical power of 0.95. Given the lack of directly comparable prior studies, a conservative effect size [25] (*d* = 0.30) was adopted as a benchmark. The final sample size of 204 participants significantly exceeds this requirement, ensuring high statistical sensitivity.

### 2.3. Exclusion and Inclusion Criteria

Participants were excluded if they had any systemic or psychiatric disorder; significant cognitive impairments or language barriers preventing questionnaire completion; current use of medications affecting anxiety or emotional states; prior dental treatment; or a need for emergency dental care.

### 2.4. Data Collection

The researcher recorded each participant’s age, gender, residence, family income, number of siblings, birth order, and parental education level. Household income was grouped into three tiers—low, middle, and high—using official monthly minimum wage as the reference threshold.

After sociodemographic data were recorded, each child completed Corah’s Dental Anxiety Scale (CDAS) and the Rosenberg Self-Esteem Scale (RSES) by themselves within the clinic’s waiting room, with no parent present. The researcher clarified the wording of specific items verbally whenever a child indicated difficulty understanding them. To minimize bias, the questionnaires were administered during the child’s first visit, before any dental treatment.

Dental anxiety was measured using the CDAS [26], a four-item instrument scored on a five-point Likert scale ranging from 1 (not anxious) to 5 (extremely anxious), with total scores ranging from 4 to 20. Higher scores reflect greater dental anxiety; a score of 12 or above suggests the presence of dental anxiety, while a score of 15 or more indicates a high level of anxiety.

Self-esteem was measured using the RSES [27], a ten-item scale comprising five positively worded and five negatively worded items, rated on a four-point Likert scale (1 = strongly disagree, 4 = strongly agree). The negatively worded items were reverse-coded before analysis, producing total scores ranging from 10 to 40, with higher scores indicating higher self-esteem.

In the present study, the Turkish adaptations of both scales, previously established as valid and reliable, were used to ensure linguistic and conceptual consistency [28,29]. We also re-assessed the internal consistency of each instrument within our sample by calculating Cronbach’s alpha. The CDAS showed high reliability (Cronbach’s α = 0.848), whereas the RSES demonstrated acceptable reliability (Cronbach’s α = 0.762). These reliability estimates were broadly comparable to those reported in the original Turkish adaptation studies (CDAS: α = 0.81 [29]; RSES: α = 0.862 [28]).

### 2.5. Statistical Analysis

The normality of the data distribution was checked using the Shapiro–Wilk test; because most variables did not meet this assumption, nonparametric methods were applied. Group comparisons were carried out with the Mann–Whitney U test for two independent groups and the Kruskal–Wallis test for three or more groups. Descriptive statistics were presented as mean ± standard deviation (SD). Spearman’s rank correlation coefficient was used to examine associations between continuous variables, including age, dental anxiety, and self-esteem scores. Independent predictors of dental anxiety were examined using a stepwise multiple linear regression. This method was selected for its exploratory value in a relatively unexplored predictor set; given the known limitations of stepwise selection, including model instability and inflated Type I error, findings should be interpreted as hypothesis-generating. Sociodemographic variables entered the model as dummy-coded predictors, with the most common or baseline category serving as the reference in each case. Model assumptions were checked via the Durbin–Watson statistic, which confirmed the absence of autocorrelation, alongside residual analysis to verify normality. All statistical analyses were conducted on IBM SPSS Statistics, version 26.0 (IBM Corp., Armonk, NY, USA) and the threshold of a *p*-value of <0.05 defined statistical significance.

## 3. Results

### 3.1. Demographic Characteristics and Scale Scores

Ages among the 204 participants ranged from 7 to 12 years, with a mean of 9.86 ± 1.61 years. Most participants lived in urban areas (62.7%), and nearly half belonged to two-child families (45.1%). Regarding parental education, most mothers (34.3%) and fathers (44.6%) were high-school graduates. In terms of socioeconomic status, 47.1% of families were classified as belonging to the middle-income group.

According to the CDAS scores, 24.5% of the children were found to be anxious (CDAS ≥ 12), and 12.5% exhibited high anxiety (CDAS ≥ 15). The overall distribution of participants and comparisons of scale scores according to demographic variables are summarized in Table 1.

There were no statistically significant differences in dental anxiety or self-esteem scores according to gender, place of residence, number of siblings, birth order, parental education level, or family income (*p* > 0.05 for all, Table 1). Although mean anxiety scores were slightly higher among children living in rural areas and among those whose parents had lower education levels, these differences were not statistically significant. Similarly, self-esteem scores were slightly higher among children from higher-income families and those whose fathers had university-level education, yet these differences were also nonsignificant (*p* > 0.05). A weak, non-significant correlation was found between age and both scale scores, as shown in Table 2.

### 3.2. Correlation Between Dental Anxiety and Self-Esteem

A moderate, negative, and statistically significant correlation was found between the total scores of the CDAS and the RSES (*r* = −0.301, *p* < 0.001), as shown in Table 3. This result indicates that higher self-esteem levels were associated with lower dental anxiety.

### 3.3. Regression Analysis

The stepwise model identified self-esteem as the sole significant independent predictor of dental anxiety within this sample (F = 18.704, *p* < 0.001; Table 4), accounting for 8% of the variance in anxiety scores (Adjusted R^2^ = 0.080, *p* < 0.001). Other variables, including age, gender, and socioeconomic status, did not significantly contribute to the model and were excluded during the stepwise procedure (*p* > 0.05 for all; Table 5).

## 4. Discussion

Dental anxiety, as a behavioral management challenge, can complicate dental treatment, delay appointments, and increase the risk of oral health problems. While poor oral health may adversely affect children’s psychosocial well-being, evaluating the role of internal psychological factors, such as self-esteem, remains a critical area of investigation. According to the CDAS results, nearly one-quarter of the participants (24.5%) exhibited dental anxiety. Reported prevalence in Turkish pediatric samples has varied considerably, ranging from 11% [30] to 25.3% [31], likely reflecting differences in assessment tools, age ranges, sampling settings, and cutoff thresholds used to define clinically significant anxiety rather than true differences in underlying population risk.

A notable finding of this research was that traditional sociodemographic variables (age, gender, socioeconomic status, etc.) showed no significant association with dental anxiety. Although several studies have suggested that age is inversely linked with dental anxiety [2,32], as older children tend to develop better coping mechanisms for dental stress [6,21], or that females exhibit higher anxiety levels [2], our current findings align with studies reporting no such correlation [11,33]. Similarly, while lower socioeconomic status is often linked to higher dental anxiety due to limited access to preventive care [7,8,13], our findings did not reveal a significant impact of family income or parental education. Given that traditional demographic factors showed no significant impact in our sample, the focus shifts toward internal psychological constructs.

Although the link between oral health and self-esteem is well-documented [18], research exploring the direct association between dental anxiety and self-esteem in children is relatively limited [19], and the existing evidence is mixed. A cross-cultural study involving preadolescents in Turkey and Finland reported only a small negative correlation between self-esteem and dental anxiety, after accounting for various psychological and maternal factors [24]. Similarly, a systematic review found that while self-esteem correlates with oral health-related quality of life, its specific link with dental anxiety was deemed insufficient to reach a definitive conclusion [14]. In a study performed among Lithuanian children, low self-esteem was found to be associated with higher dental anxiety in both boys and girls, with the relationship being more pronounced among older children [34]. In our study, we aimed to help clarify these inconsistent findings using multivariable regression to account for the simultaneous contribution of sociodemographic variables. In our sample, regression analysis confirmed self-esteem as the only significant independent predictor of dental anxiety (*p* < 0.001). Considering that dental fear has been associated with complex etiologic factors—such as past traumatic experiences, parental anxiety [32], and general temperament [5]—the finding that a single psychological construct independently accounts for 8% of the variance (Adjusted R^2^ = 0.080, Table 4) is noteworthy. Nevertheless, the modest effect size indicates that self-esteem alone should not be regarded as a primary clinical marker of dental anxiety; rather, it may serve as one component within a broader behavioral assessment that also considers prior dental experiences or situational factors during clinical encounters. These results indicate that children with higher self-esteem tended to report lower levels of dental anxiety. The identification of self-esteem as an independent, if modest, predictor suggests that a child’s psychological profile may warrant further investigation as a potential complement to established sociodemographic factors in behavioral management research, though whether such assessment translates into improved clinical outcomes remains to be tested.

This study has a few limitations. Since the research was performed at a single center, the findings may not generalize to all children. In addition, information on children’s oral health status (e.g., caries experience or treatment needs) and parental dental anxiety was not collected. As each of these factors is a known correlate of pediatric dental anxiety, their absence represents unmeasured confounders that may have influenced the observed self-esteem–anxiety relationship. Moreover, as this was a cross-sectional study performed within a clinical environment, only children who presented for dental care could be enrolled, which may have introduced selection bias by excluding children who avoid dental visits altogether—potentially those with the highest levels of dental anxiety. Although the sample size was sufficient for the main goals, there were few children from rural areas. This may have made it harder to detect the impact of where children live; furthermore, urban and rural environments may differ in healthcare access, cultural attitudes toward dental care, and parenting practices, all of which could modulate the self-esteem–dental anxiety relationship in ways this study was not powered to detect, and future studies with balanced urban–rural representation are needed to clarify whether this association holds consistently across settings.

Methodologically, the use of stepwise selection carries inherent risks of model instability and Type I error inflation; however, the small number of candidate predictors in this analysis limits the associated overfitting risk. Future studies with larger predictor sets may benefit from hierarchical or penalized approaches, which offer greater stability at the cost of exploratory flexibility. Finally, the cross-sectional design of this study does not permit causal conclusions, and it remains obscure whether low self-esteem precedes dental anxiety, is a consequence of it, or reflects a bidirectional relationship; longitudinal studies are needed to clarify the temporal and potentially reciprocal nature of this association.

## 5. Conclusions

This study suggests that a child’s psychological profile, particularly self-esteem, is associated with dental anxiety. From a practical standpoint, clinicians may consider briefly incorporating self-esteem-related observations (e.g., a child’s confidence and comfort during interaction) into behavioral management alongside well-established methods including tell-show-do and positive reinforcement. For future research, prospective, multi-center studies involving diverse populations of children that assess self-esteem alongside prior dental experience, parental anxiety, and oral health status are needed to clarify the temporal and potentially causal nature of this association, as well as the broader psychological and environmental factors underlying dental fear.

## Figures and Tables

**Table 1:** Distribution of sociodemographic variables and their association with dental anxiety and self-esteem scores (*n* = 204).

Variable	*n* (%)	Dental Anxiety (Mean ± SD)	*p*-Value	Self-Esteem (Mean ± SD)	*p*-Value
**Gender**	Female: 113 (55.4)	8.92 ± 3.77	0.403 *	32.83 ± 4.31	0.778 *
	Male: 91 (44.6)	8.68 ± 4.32		33.02 ± 4.33	
**Residence**	Village: 7 (3.4)	11.71 ± 6.34	0.152 **	32.29 ± 5.35	0.696 **
	District: 69 (33.8)	8.10 ± 3.57		33.32 ± 4.09	
	City: 128 (62.7)	9.04 ± 4.04		32.73 ± 4.39	
**Number of children in family**	1: 15 (7.4)	7.60 ± 4.40	0.070 **	32.73 ± 3.15	0.208 **
	2: 92 (45.1)	9.27 ± 4.16		32.39 ± 4.39	
	3: 76 (37.3)	8.26 ± 3.80		33.61 ± 4.19	
	≥4: 21 (10.3)	9.47 ± 3.50		33.11 ± 4.97	
**Birth order**	1st: 78 (38.2)	8.49 ± 3.78	0.313 **	32.85 ± 4.37	0.455 **
	2nd: 80 (39.2)	9.30 ± 4.25		32.60 ± 4.40	
	≥3rd: 46 (22.6)	8.32 ± 3.99		33.43 ± 4.05	
**Maternal education**	≤Primary: 52 (25.5)	8.42 ± 3.46	0.588 **	33.26 ± 4.94	0.741 **
	Secondary: 43 (21.1)	8.51 ± 3.91		32.28 ± 4.44	
	High school: 70 (34.3)	9.20 ± 4.10		32.86 ± 4.05	
	≥University: 39 (19.1)	8.89 ± 4.36		33.08 ± 3.92	
**Paternal education**	≤Primary: 64 (31.4)	8.79 ± 3.85	0.479 **	33.15 ± 4.91	0.157 **
	Secondary: 31 (15.2)	9.42 ± 3.99		33.29 ± 4.29	
	High school: 91 (44.6)	8.78 ± 4.00		32.12 ± 4.22	
	≥University: 49 (24.0)	8.77 ± 4.09		34.03 ± 4.16	
**Monthly** **income**	Low income: 73 (35.8)	8.84 ± 4.01	0.873 **	33.44 ± 4.13	0.124 **
	Middle income: 96 (47.1)	8.90 ± 4.06		32.24 ± 4.45	
	High income: 35 (17.2)	8.54 ± 4.02		33.69 ± 4.11	

Note: Continuous variables, mean ± SD; categorical variables, n (%). * Mann–Whitney U test; ** Kruskal–Wallis test; p < 0.05 considered significant.

**Table 2:** Spearman correlation analysis between participants’ age and dental anxiety and self-esteem scores.

Variable	Age
CDAS	*r* = −0.015, *p* = 0.834
RSES	*r* = 0.071, *p* = 0.313

*Note: r: rank correlation coefficient; p < 0.05 significant difference*.

**Table 3:** Spearman correlation analysis between Corah’s Dental Anxiety Scale (CDAS) and Rosenberg Self-Esteem Scale (RSES) scores.

*Variable*	*RSES*
CDAS	*r* = −0.301, *p* < 0.001

*Note: r: rank correlation coefficient; p < 0.05 significant difference*.

**Table 4:** Results of the stepwise multiple linear regression analysis predicting dental anxiety (CDAS) scores.

Independent Variables	B	SE	β	*p*-Value	95% CI
Intercept	17.748	2.083	-	<0.001	[13.640, 21.856]
Self-Esteem (RSES)	−0.271	0.063	−0.291	<0.001	[−0.395, −0.148]

*Note: R = 0.291, R^2^= 0.085, Adjusted R^2^ = 0.080; F (1, 202) = 18.704, p < 0.001. B: Unstandardized coefficient, SE: Standard error, β: Standardized coefficient, CI: Confidence Interval. See Table 5 for variables excluded from the final model*.

**Table 5:** Variables excluded from the final stepwise regression model.

Variable	β (Beta in)	t	*p*
Age	*−0.043*	*−0.631*	*0.529*
Gender (female)	*0.023*	*0.345*	*0.731*
Residence (district)	*−0.108*	*−1.608*	*0.109*
Residence (city)	*0.057*	*0.847*	*0.398*
Number of children (2)	*0.072*	*1.067*	*0.287*
Number of children (3)	*−0.071*	*−1.047*	*0.296*
Number of children (≥4)	*0.100*	*1.485*	*0.139*
Birth order (2nd)	*0.081*	*1.195*	*0.233*
Birth order (≥3rd)	*−0.038*	*−0.560*	*0.576*
Maternal education (secondary)	*−0.062*	*−0.913*	*0.363*
Maternal education (high school)	*0.067*	*0.992*	*0.323*
Maternal education (≥university)	*0.052*	*0.765*	*0.445*
Paternal education (secondary)	*0.075*	*1.111*	*0.268*
Paternal education (high school)	*−0.057*	*−0.841*	*0.401*
Paternal education (≥university)	*0.042*	*0.615*	*0.539*
Income (middle)	*−0.024*	*−0.359*	*0.720*
Income (high)	*−0.007*	*−0.105*	*0.917*

*Note: β (Beta in): standardized coefficient if the variable were entered into the model. Reference categories: male (gender), village (residence), 1 child (number of children), 1st (birth order), ≤primary school (maternal and paternal education), low income (monthly income)*.

## Data Availability

The data presented in this study are available on reasonable request from the corresponding author. The data are not publicly available due to restrictions, e.g., privacy or ethical.

## Abbreviations

The following abbreviations are used in this manuscript:

CDAS	Corah’s Dental Anxiety Scale
RSES	Rosenberg Self-Esteem Scale
STROBE	Strengthening the Reporting of Observational Studies in Epidemiology
